# Serum resolvin E1 levels and its relationship with thyroid autoimmunity in Hashimoto’s thyroiditis: a preliminary study

**DOI:** 10.1186/s12902-021-00730-9

**Published:** 2021-04-13

**Authors:** Jing Song, Rongxin Sun, Yuanyuan Zhang, Jing Ke, Dong Zhao

**Affiliations:** Beijing Key Laboratory of Diabetes Research and Care, Center for Endocrine Metabolism and Immune Diseases, Lu He Hospital Capital Medical University, Beijing, China

**Keywords:** Hashimoto's thyroiditis, Resolvins, Serum, RVE1

## Abstract

**Background:**

Omega-3 polyunsaturated fatty acids (PUFAs) produce lipid mediators with both anti-inflammatory and pro-resolution properties, including resolvins. The purpose of this study was to detect serum resolvin E1 (RVE1) levels in Hashimoto’s thyroiditis (HT) patients and healthy controls (HCs) and to evaluate the relationship of RVE1 with thyroid autoimmunity.

**Methods:**

A total of 57 participants were recruited, including 30 untreated HT patients and 27 age- and sex‐matched HCs. The levels of RVE1 in serum were measured via enzyme-linked immunosorbent assay (ELISA). An electrochemiluminescence immunoassay was used for the measurement of thyroid-stimulating hormone (TSH), total T4 (TT4), TT3, free T4 (FT4), FT3, anti-thyroid peroxidase antibody (TPOAb) and anti-thyroglobulin antibody (TgAb) levels. Hemogram tests and routine biochemical analyses were performed on each sample.

**Results:**

The serum level of RVE1 of HT patients (24.09, 15.76–34.38 pg/mL) was significantly lower than that of healthy controls (28.51, 20.76–51.23 pg/mL) (*P* = 0.027). RVE1 levels showed a downward trend with increasing TgAb levels (P for trend = 0.001). Multivariable ordinal logistic regression analysis showed that RVE1 levels were negatively correlated with increasing TgAb levels in both the unadjusted (OR = 0.9446, 95 % CI = 0.9111–0.9782, *P* = 0.002) and adjusted models (OR = 0.9380, 95 % CI = 0.8967–0.9811, *P* = 0.005).

**Conclusions:**

Decreased RVE1 levels might be a sign that HT is associated with inflammatory resolution dysfunction. RVE1 may serve as a protective factor against increased TgAb levels.

## Introduction

Hashimoto’s thyroiditis (HT), which is also known as chronic lymphocytic thyroiditis, is now considered the most common autoimmune disease. HT is characterized by the production of autoantibodies against thyroid peroxidase (TPO) and thyroglobulin (Tg) in the serum and the presence of infiltrating lymphocytes in the thyroid. Most HT patients will eventually develop primary hypothyroidism [1]. A cross-sectional study of 31 provinces in mainland China showed that the anti-TPO antibody (TPOAb) and the anti-Tg antibody (TgAb) positivity rates in Chinese adults were 10.19 and 9.70 %, respectively [2]. Recent studies have confirmed that TgAb and TPOAb can increase the risk of thyroid cancer and thyroid nodules [3, 4]. High levels of thyroid autoantibodies not only cause hypothyroidism but also are an essential factor in HT disease progression. Studying euthyroid HT patients revealed that high levels of thyroid autoantibodies can lead to a lower quality of life score and vestibular dysfunction [5, 6]. Given that euthyroid HT patients already bear the above burden, early diagnosis and intervention are particularly important. HT is currently considered an autoimmune disease affected by genetic and environmental factors. Among these factors, some nutritional factors have been confirmed to be related to HT, such as selenium and vitamin D [7, 8]. Selenium supplementation can significantly reduce thyroid autoantibody levels in patients with HT [9]; however, the clinical efficacy of selenium supplementation is controversial. Therefore, it is necessary to further study nutritional factors that have a protective effect on HT.

Chronic inflammation is the underlying mechanism of disease progression in many autoimmune diseases. Recent research suggests that the progression of chronic inflammatory diseases may be due to impaired resolution and has revealed the protective effect of endogenous lipid mediators [10]. Once thought to be a passive process, acute inflammation has now been shown to involve an active inflammatory resolution process and promote the restoration of inflamed tissue to a steady state [11]. The resolution process involves the following steps: remove the harmful substances that trigger the inflammatory response, stop the synthesis of proinflammatory mediators and promote their decomposition and metabolism, and then terminate leukocyte recruitment. Neutrophils and lymphocytes undergo apoptosis or necrosis and are finally cleared by macrophages [12]. In addition, the resolution of inflammation is an active process induced via endogenous specialized pro-resolving mediators (SPMs). SPMs are downstream derivatives of polyunsaturated fatty acids (PUFAs), which include resolvins, maresins, lipoxins and protectins [13].

Resolvins include E series (RVE1-RVE3) and D series (RVD1-RVD6), which are produced from eicosapentaenoic acid (EPA) and docosahexaenoic acid (DHA), respectively. Among them, RVE1 triggers all aspects of the pro-resolution cascade from inhibiting lymphocyte aggregation at the inflammation site to inflammatory fragment efferocytosis or removal [14]. Notably, RVE1 has a protective effect in many chronic inflammatory disease models. RVE1 induces the expression of its specific receptor chemR23 on the surface of dendritic cells and inhibits the release of IL-12, thus hindering inflammation progression [15]. RVE1 inhibits bone resorption in the inflammatory environment by controlling the ratio of RANKL/OPG and downstream genetic factors [16]. Additionally, omega-3 PUFAs are upstream substances of RVE1 and have a therapeutic effect on some autoimmune diseases. EPA significantly alleviates the disease progression of experimental autoimmune encephalomyelitis (EAE) [17]. The application of omega-3 PUFAs and fish oil has a beneficial effect on reducing the recurrence rate and inflammatory indicator levels and improving the quality of life in patients with multiple sclerosis [18]. Supplementation with omega-3 fatty acids (EPA and DHA) improves type 1 diabetes by regulating autoimmunity and suppressing inflammation [19, 20]. Similarly, omega-3 PUFAs display potential protective effects in thyroid disease; a case report described a female HT patient who refused thyroid replacement therapy but was eventually cured clinically through dietary management (including omega-3 PUFAs) [21]. Therefore, we infer that the omega-3 PUFA downstream derivative RVE1 may also play an important role in the development of HT.

To date, no studies have investigated the changes in RVE1 in HT patients and HCs. Therefore, this study mainly measured the serum RVE1 levels of HT patients and HCs and further analysed the correlations between RVE1 and thyroid autoantibodies and other clinical indicators.

## Methods

### Study groups

We recruited 57 participants from the endocrinology clinic of Beijing Luhe Hospital, namely, 30 untreated HT patients and 27 age- and sex‐matched HCs. All participants had no history of other autoimmune diseases, any acute or chronic illness, smoking, or alcohol intake and no current use of medications or pregnancy. The diagnostic criteria for HT were diffuse goitre, TPOAb and/or TgAb positivity, and B ultrasound examination revealing uneven hypoechoic changes and diffuse thyroid enlargement. All participants fasted, and blood samples were collected between 8 and 10 AM.

An electrochemiluminescence immunoassay was used to detect serum TgAb, TPOAb, free T3 (FT3), FT4, total T3 (TT3), TT4, and TSH levels. Moreover, routine blood examination (neutrophil, leukocyte, haemoglobin, and platelet levels) and biochemical detection were performed on each sample. Biochemical detection included analysis of liver function markers (aspartate aminotransferase (AST) and alanine aminotransferase (ALT)), albumin, blood glucose, electrolytes (sodium and potassium), creatinine, blood urea nitrogen (BUN), total cholesterol (TC), high-density lipoprotein (HDL), triglycerides (TG), low-density lipoprotein (LDL), C-reactive protein (CRP) and uric acid.

### Assay of serum RVE1 levels

A RVE1 ELISA kit (Signalway Antibody, USA) was applied to measure serum RVE1 levels according to the manufacturer’s instructions. All serum samples were assayed on the same day. The inter-assay and intra-assay coefficients of variation (CVs) for RVE1 were < 10 %.

### Statistical analysis

SPSS 23.0 software (SPSS, Chicago, IL, USA) was used for the statistical analysis. Normally distributed data are expressed as the mean ± standard deviation (M ± SD), and comparisons were performed with independent samples t tests. Data with abnormal distributions are expressed as the median and interquartile range (IQR), and the distributions between groups were compared using the nonparametric Mann-Whitney U test. The Spearman correlation coefficient (r) was used to analyse the correlation between RVE1 and other variables.

According to the interquartile range, TPOAb levels were divided into Q1: <13.6 U/mL, Q2: 13.6–10.6 U/mL, Q3: 10.6–431 U/mL, and Q4: >431 U/mL and TgAb levels were divided into Q1: <12.9 U/mL, Q2: 12.9–79.1 U/mL, Q3: 79.1–361 U/mL, and Q4: >361 U/mL. Multivariable ordinal logistic regression models were used to evaluate the relationships between RVE1 and thyroid autoantibodies. The Jonckheere-Terpstra test was used to evaluate trends. All *P*-values were two-sided, and *P* < 0.05 was defined as statistically significant.

## Results

A total of 57 participants (30 HT patients and 27 HCs) were included in our research. As shown in Table 1, no significant difference in age or sex was noted between HT patients and HCs. The levels of thyroid-specific antibodies (TPOAb and TgAb) in the HT group were significantly greater than those in the HCs group (*P* < 0.001 for all). No differences in thyroid parameters (TSH, TT4, FT4, TT3, and FT3), blood cells and routine biochemical indicators were noted between the two groups (*P* > 0.05 for all).

**Table 1:** Clinical and demographic characteristics of the HT patients and HCs

	HT (*n* = 30)	HCs (*n* = 27)	*P* value
Age (years)^a^	40.27 ± 14.43	50.22 ± 18.71	0.096
Sex (F/M)	28/2	26/1	0.997
TT3 (ng/mL)^b^	0.98 (0.86–1.12)	1.02 (0.89–1.15)	0.497
TT4 (µg/dL)^b^	6.65 (5.78–7.94)	6.91 (5.82–7.58)	0.767
FT3 (pg/mL)^b^	2.95 (2.74–3.10)	3.08 (2.81–3.35)	0.096
FT4 (ng/dL)^b^	1.17 (1.02–1.32)	1.22 (1.16–1.33)	0.240
TSH (uIU/mL)^b^	2.62 (1.95–3.05)	2.38 (1.54–2.94)	0.178
TgAb (U/mL)^b^	360.00 (151.75–400.00)	12.80 (10.80–14.10)	< 0.001
TPOAb (U/mL)^b^	417.00 (132.00-522.75)	13.60 (9.00-16.69)	< 0.001
Leukocyte levels (×10^9^/L)^b^	6.33 (5.46–6.95)	6.31 (5.36–7.90)	0.653
Hemoglobin (g/L)^b^	138.50 (132.00-152.00)	141.00 (134.00-146.00)	0.872
Platelet count (×10^9^/L)^b^	249.50 (185.00-266.50)	239.00 (219.00-287.00)	0.602
Albumin (g/L)^b^	46.30 (44.50-47.75)	45.40 (44.30–47.20)	0.506
ALT (U/L)^b^	19.00 (15.75-34.00)	19.00 (9.00–28.00)	0.627
AST (U/L)^b^	18.50 (14.75-24.00)	16.00 (15.00–24.00)	0.602
Serum potassium (mmol/L)^b^	4.32 (3.95–4.73)	4.17 (3.90–4.41)	0.448
Serum sodium (mmol/L)^b^	139.00 (138.00-140.00)	140.00 (139.00-141.00)	0.068
Blood glucose (mmol/L)^b^	5.69 (5.26–6.08)	5.39 (5.21–5.98)	0.650
Creatinine (µmol/L)^b^	63.00 (54.50–76.50)	65.00 (63.00–80.00)	0.472
BUN (mmol/L)^b^	4.30 (3.82–5.34)	4.28 (3.32–5.47)	0.545
Uric acid (µmol/L)^b^	331.00 (252.50-374.50)	297.00 (259.00-323.00)	0.238
TG (mmol/L)^b^	1.67 (1.14–2.10)	1.31 (0.80–1.72)	0.208
TC (mmol/L)^b^	5.04 (4.73–5.55)	4.41 (4.12–5.35)	0.191
HDL (mmol/L)^b^	1.26 (1.02–1.63)	1.33 (1.14–1.77)	0.404
LDL (mmol/L)^b^	3.05 (2.86–3.55)	2.69 (2.19–3.07)	0.091
CRP (mg/L)^b^	0.91 (0.78–2.52)	1.73 (0.36–2.92)	0.997

*HT* Hashimoto’s thyroiditis; *TT4* total T4; *TT3* total T3; *FT3* free T3; *FT4* free T4; *TSH* thyroid-stimulating hormone; *TPOAb* anti-thyroperoxidase antibody; *TgAb* anti-thyroglobulin antibody; *ALT* alanine aminotransferase; *AST* aspartate aminotransferase; *BUN* blood urea nitrogen; *TG* triglyceride, *TC* total cholesterol; *HDL* high-density lipoprotein; *LDL* low-density lipoprotein; *CRP* C-reactive protein

The standard reference range as follows: TgAb, 0-115 U/mL; TPOAb, 0‐34 U/mL; TT3, 0.61‐1.77 ng/mL; TT4, 5.13‐14.06 ug/dL; FT3, 3.10‐6.80 pg/mL; FT4, 12.00‐22.0 ng/dL; TSH, 0.027‐4.20 uIU/mL

^a^The results are expressed as means ± SD, ^b^The results are presented as medians (interquartile range). The P values were obtained through statistical analyses using independent samples t-tests or Mann-Whitney U tests

**Fig. 1:**
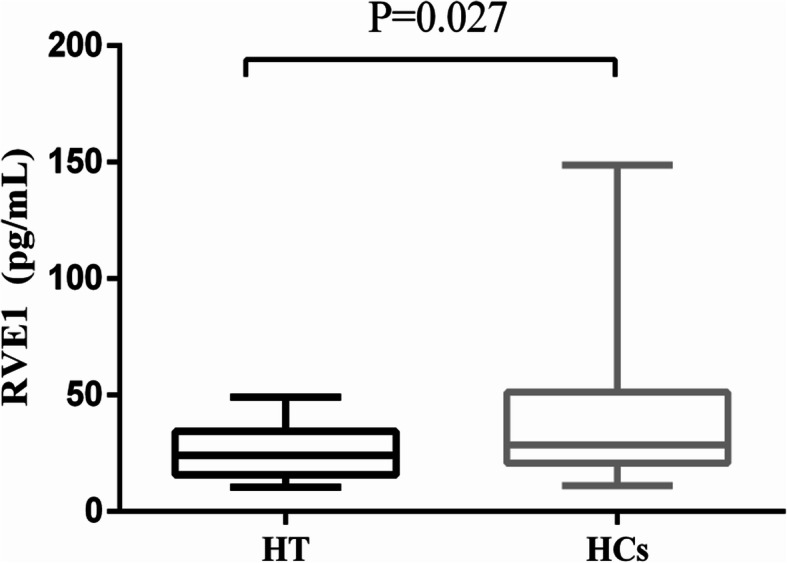
Comparison of serum resolvin E1 (RVE1) levels in patients with Hashimoto’s thyroiditis (*n* = 30) and healthy controls (*n* = 27). Data were expressed as median and interquartile intervals. HT: Hashimoto’s thyroiditis. HCs: Healthy controls

The serum level of RVE1 of HT patients (24.09, 15.76–34.38 pg/mL) was significantly reduced compared with those of healthy controls (28.51, 20.76–51.23 pg/mL) (*P* = 0.027) (Fig. 1).

We performed Spearman correlation analysis to assess the relationships between serum RVE1 and other variables for all participants. RVE1 was significantly negatively correlated with TgAb (*r*=-0.426, *P* = 0.001), TT3 (*r*=-0.348, *P* = 0.008) and FT3 (*r*=-0.339, *P* = 0.010) (Table 2).

**Table 2:** Spearman correlation analysis between RVE1 levels and clinical characteristics in all the participants

	Correlation coefficient	*P* value
TT3 (ng/mL)	-0.348	0.008*
TT4 (µg/dL)	0.011	0.934
FT3 (pg/mL)	-0.339	0.010*
FT4 (ng/dL)	0.146	0.278
TSH (uIU/mL)	-0.019	0.891
TgAb (U/mL)	-0.426	0.001*
TPOAb (U/mL)	-0.244	0.067
Leukocyte levels (×10^9^/L)	-0.083	0.645
Hemoglobin (g/L)	-0.101	0.574
Platelet count (×10^9^/L)	-0.133	0.461
Albumin (g/L)	0.059	0.743
ALT (U/L)	-0.001	0.996
AST (U/L)	-0.153	0.396
Serum potassium (mmol/L)	-0.157	0.391
Serum sodium (mmol/L)	0.273	0.130
Blood glucose (mmol/L)	0.249	0.169
Creatinine (µmol/L)	0.038	0.836
BUN (mmol/L)	-0.207	0.256
Uric acid (µmol/L)	-0.094	0.611
TG (mmol/L)	0.167	0.395
TC (mmol/L)	-0.161	0.413
HDL (mmol/L)	0.062	0.752
LDL (mmol/L)	-0.176	0.371
CRP (mg/L)	-0.999	0.645

*TT4* total T4; *TT3* total T3; *FT3* free T3; *FT4* free T4; *TSH* thyroid-stimulating hormone; *TPOAb* anti-thyroperoxidase antibody; *TgAb* anti-thyroglobulin antibody; *ALT* alanine aminotransferase; *AST* aspartate aminotransferase; *BUN* blood urea nitrogen; *TG* triglyceride, *TC* total cholesterol; *HDL* high-density lipoprotein; *LDL* low-density lipoprotein; *CRP* C-reactive protein; *P < 0.05

To evaluate the relationship between RVE1 and thyroid autoantibodies, we divided the TPOAb and TgAb levels into four categories according to the interquartile range. As shown in Table 3, the RVE1 level showed a downward trend with increasing TgAb levels (P for trend = 0.001). The RVE1 content in the Q4 (TgAb > 361 IU/mL) group (19.21, 13.81–27.34 pg/mL) was significantly lower than that in the Q1 (TgAb < 34 IU/mL) group (37.70, 24.66–99.16 pg/mL) (*P* = 0.005). Multivariable ordinal logistic regression analysis showed that RVE1 was negatively correlated with TgAb in both the unadjusted (OR = 0.9446, 95 % CI = 0.9111–0.9782, *P* = 0.002) and adjusted models (OR = 0.9475, 95 % CI = 0.9166–0.9792, *P* = 0.001 and OR = 0.9380, 95 % CI = 0.8967–0.9811, 

*P* = 0.005). As shown in Table 4, we also assessed the relationship between RVE1 and TPOAb levels. As TPOAb levels increased, RVE1 levels exhibited an inverted U-shaped trend (P for trend = 0.036). The RVE1 content reached the highest value in the Q2 group. Moreover, the RVE1 content in the Q4 group (19.21, 15.11–26.01 pg/mL) was significantly lower than that in the Q2 group (31.39, 23.79–62.31 pg/mL) (*P* = 0.019). Multivariable ordinal logistic regression analysis showed that RVE1 was negatively correlated with TPOAb in both the unadjusted (OR = 0.9762, 95 % CI = 0.9559–0.9970, *P* = 0.028) and adjusted models (OR = 0.9772, 95 % CI = 0.9567–0.9980, *P* = 0.011, adjusted for age and sex). However, when the model was adjusted for age, sex, and TT3, TT4, TSH, FT3, FT4, and TgAb levels, RVE1 levels showed no significant correlation with increasing TPOAb levels (OR = 0.9860, 95 % CI = 0.9627-1.010, *P* = 0.244).

**Table 3:** Multivariable ordinal logistic regression to investigate the association between RVE1 and TgAb

Interquartile range of TgAb	Q1 (< 12.9 U/mL) (*n* = 14)	Q2 (12.9–79.1 U/mL) (*n* = 14)	Q3 (79.1–361 U/mL) (*n* = 15)	Q4 (> 361 U/mL) (*n* = 14)	
^b^ RVE1 Levels	37.70 (24.66–99.16)	26.46 (18.91–34.87)	28.51 (19.53–37.61)	19.21 (13.81–27.34) *	^a^ P for trend = 0.001
Multivariable ordinal logistic regression					
	β (95 % CI)		OR (95 % CI)		*P* value
Model 1	-0.057 (-0.093, -0.022)		0.9446 (0.9111, 0.9782)		0.002
Model 2	-0.054 (-0.087, -0.021)		0.9475 (0.9166, 0.9792)		0.001
Model 3	-0.064 (-0.109, -0.019)		0.9380 (0.8967, 0.9811)		0.005

According to the interquartile range, TgAb was divided into four groups: Q1: the first TgAb quartile group (TgAb < 12.9 U/mL), Q2: the second TgAb quartile group (12.9–79.1 U/mL), Q3: the third TgAb quartile group (79.1–361 U/mL), Q4: the fourth TgAb quartile group (> 361 U/mL)

^a^The Jonckheere-Terpstra test was used to evaluate trends of RVE1 levels when the TgAb level increased

^b^ Results are expressed as medians (interquartile ranges). The Kruskal-Wallis test was used to detect differences in RVE1 levels among the four groups, and Bonferroni‐adjusted *P* values were used, *P = 0.005 vs. Q1 group

Multivariable ordinal logistic regression models were used to evaluate relationships between RVE1 and increasing TgAb levels. Model 1 was not adjusted for other variables; Model 2 was adjusted for age and sex; Model 3 was adjusted for age, sex, TT3, TT4, TSH, FT3, FT4 and TPOAB. RVE1, resolvin E1; OR, odds ratio; 95 % CI, 95 % confidence interval

**Table 4:** Multivariable ordinal logistic regression to investigate the association between RVE1 and TPOAb

Interquartile range of TPOAb	Q1 (< 13.6 U/mL) (*n* = 14)	Q2 (13.6–106 U/mL) (*n* = 14)	Q3 (106–431 U/mL) (*n* = 15)	Q4 (> 431 U/mL) (*n* = 14)	
^b^ RVE1 Levels	26.16 (19.53–38.64)	31.39 (23.79–62.31)	28.51 (17.03–40.38)	19.21 (15.11–26.01) *	^a^ P for trend = 0.036
Multivariable ordinal logistic regression					
	β (95 % CI)		OR (95 % CI)		*P* value
Model 1	-0.024 (-0.045, -0.003)		0.9762 (0.9559, 0.9970)		0.028
Model 2	-0.023 (-0.044, -0.002)		0.9772 (0.9569, 0.9980)		0.011
Model 3	-0.014 (-0.038, 0.010)		0.9860 (0.9627, 1.010)		0.244

According to the interquartile range, TPOAb was divided into four groups: Q1: the first TPOAb quartile group (TgAb < 13.6 U/mL), Q2: the second TPOAb quartile group (13.6–10.6 U/mL), Q3: the third TPOAb quartile group (10.6–431 U/mL), Q4: the fourth TPOAb quartile group (> 431 U/mL)

^a^The Jonckheere-Terpstra test was used to evaluate trends in RVE1 levels when TPOAb levels increased

^b^ Results are expressed as medians (interquartile ranges). The Kruskal-Wallis test was used to detect differences in RVE1 levels among the four groups, and Bonferroni‐adjusted *P* values were used, *p = 0.019 vs. Q2 group

Multivariable ordinal logistic regression models were used to evaluate relationships between RVE1 and increasing TPOAb levels. Model 1 was not adjusted for other variables; Model 2 was adjusted for age and sex; Model 3 was adjusted for age, sex, TT3, TT4, TSH, FT3, FT4 and TPOAB. RVE1, resolvin E1; OR, odds ratio; 95 % CI, 95 % confidence interval

## Discussion

To the best of our knowledge, this is the first study investigating serum RVE1 levels in HT patients. In the present study, we found that serum RVE1 levels in the HT group was significantly lower than that in the control group, potentially indicating dysregulation of inflammation resolution in HT patients.

Omega-3 PUFAs produce lipid mediators with both anti-inflammatory and pro-resolution properties, including resolvins [22]. The anti-inflammatory actions indicate that pro-inflammatory mediators are suppressed, whereas pro-resolution actions indicate activation of the inflammation termination process, such as apoptotic cell removal by macrophages [23]. Dysregulation of resolution increases the risk of autoimmune diseases. Although anti-inflammatory drugs can improve the symptoms of autoimmune diseases, they cannot cure the disease and are even ineffective in most patients. Therefore, the combination of anti-inflammation and pro-resolution effects may represent a superior treatment method. Moreover, pro-resolution pathways themselves do not increase the body’s susceptibility to infection [24]. Omega-3 PUFAs have shown therapeutic potential in some autoimmune diseases, including rheumatoid arthritis (RA), systemic lupus erythematosus (SLE), and type 1 diabetes mellitus (T1DM) [25]. After treatment with omega-3 PUFAs in patients with inflammatory arthritis, RVE1 levels in the knee effusion and plasma were significantly higher than that in HCs, suggesting that RVE1 may be a mediator of omega-3 PUFAs [26]. In the present study, the serum RVE1 levels in HT patients were significantly lower than those in HCs. We inferred that this difference might indicate that HT is associated with inflammatory resolution dysfunction. Similarly, RVE1 is also a marker of inflammatory resolution defects in other diseases, such as cardiovascular diseases [27], periodontitis [28], and type 2 diabetes mellitus [29].

In this study, we also analysed the correlation between levels of RVE1 and thyroid autoantibodies. The HT patients recruited in our study were euthyroid, so the interference of thyroid hormones can be excluded. Spearman correlation analysis showed that RVE1 levels were negatively correlated with TgAb levels (*r*=-0.426, *P* = 0.001). As the TgAb level increased, the RVE1 content showed a decreasing trend (P for trend = 0.001). Moreover, multivariable ordinal logistic regression analysis showed that RVE1 was negatively correlated with TgAb in both the unadjusted (OR = 0.9446, 95 % CI = 0.9111–0.9782, *P* = 0.002) and adjusted models (OR = 0.9380, 95 % CI = 0.8967–0.9811, *P* = 0.005). We also assessed the relationship between RVE1 and TPOAb levels. As TPOAb levels increased, the RVE1 levels showed an inverted U-shaped trend (P for trend = 0.036). However, when the logistic model was adjusted for age, sex, and TT3, TT4, TSH, FT3, FT4, and TgAb levels, RVE1 levels showed no significant correlation with increasing TPOAb levels (OR = 0.9860, 95 % CI = 0.9627-1.010, *P* = 0.244). Although it is currently believed that TgAb is not as specific and sensitive as TPOAb, some studies have confirmed that TgAb and TPOAb may represent two different aspects of thyroid autoimmunity. TgAb represents the initial or innate immune response, and TPOAb represents the later adaptive immune response [1]. Studies have confirmed a significant positive correlation between TgAb and clinical symptoms (fragile hair, face oedema, oedema of the eyes and harsh voice) in untreated HT patients [29]. Therefore, we hypothesize that RVE1 may serve as a protective factor against increased TgAb levels. However, further research is needed to verify the relationship between RVE1 and TgAb.

A recent study confirmed that topical application of RVE1 alleviates inflammation-induced tissue damage and reduce bone loss in an animal model of periodontitis [30]. Similarly, RVE1 administration reverses experimental periodontitis and malnutrition [31]. RVE1 inhibits the activation of dental pulp fibroblasts in a ChemR23-dependent manner and inflammation in the early stages of pulpitis [32]. The pathogeneses of autoimmune thyroid disease and periodontitis are different. The former is an autoimmune-driven disease, and the latter is an infection-driven disease. However, the two diseases have many common pathological and immunological characteristics, such as autoimmune antibodies, apoptosis, inflammation, and oxidative stress. Therefore, we infer that RVE1 may also have a protective effect on HT [33]. Further longitudinal studies are needed to verify our hypothesis. In some other diseases, the application of RVE1 has also been shown to promote inflammation resolution. RVE1 reduces airway responsiveness and inflammation in asthmatic mice [34]. Moreover, RVE1 reduces neutrophil infiltration, pro-inflammatory cytokine expression, and inflammatory pain [35]. Oral or topical application of RVE1 prevents vascular inflammation and arteriosclerosis and reduces systemic CRP levels [36]. RVE1 reduces the levels of lipopolysaccharide (LPS)-induced pro-inflammatory factors (IL-8, MCP-1) in isolated human pancreatic islets and exhibits antiapoptotic effects in a pro-inflammatory environment [37].

There are some limitations in our research. One major limitation was its cross-sectional design, as we could not observe whether RVE1 undergoes dynamic alterations related to changes in thyroid autoimmunity and thyroid function. In view of our small sample size, a follow-up longitudinal study with a larger sample size is needed to verify the role of RVE1 in HT development.

## Conclusions

Our study showed for the first time that serum RVE1 levels in HT patients were significantly reduced. Moreover, RVE1 may protect against increased TgAb levels.

## Data Availability

The datasets used and/or analysed during the current study are available from the corresponding author on reasonable request.

## Abbreviations

HT: Hashimoto’s thyroiditis
Tg: Thyroglobulin
TPO: Thyroid peroxidase
TPOAb: Anti-TPO antibody
TgAb: Anti-Tg antibody
SPMs: Specialized pro-resolving mediators
PUFAs: Polyunsaturated fatty acids
EPA: Eicosapentaenoic acid
